# Epstein-Barr Virus Neurologic Complications

**DOI:** 10.15844/pedneurbriefs-29-11-7

**Published:** 2015-11

**Authors:** J. Gordon Millichap

**Affiliations:** 1 Division of Neurology, Ann & Robert H. Lurie Children's Hospital of Chicago, Chicago, IL; 2 Departments of Pediatrics and Neurology, Northwestern University Feinberg School of Medicine, Chicago, IL

**Keywords:** Epstein-Barr Virus, Infectious Mononucleosis, Encephalitis, Cerebellitis, Myeloradiculitis, Children

## Abstract

Investigators at the Karol Marcinkowski University of Medical Sciences, Poznan, Poland, analyzed the records of 194 children diagnosed with Epstein-Barr virus infection and having the viral capsid antigen IgM antibody.

Investigators at the Karol Marcinkowski University of Medical Sciences, Poznan, Poland, analyzed the records of 194 children diagnosed with Epstein-Barr virus infection and having the viral capsid antigen IgM antibody. The most common symptoms of EB infection were drowsiness, and lymphadenopathy (in 30%) of children with neuroinfections, hepatomegaly (in 47.4%), and splenomegaly (in 41.7%). The incidence of neurologic complications was 5.2%. Patients with severe neurologic complications underwent MRI (6 abnormal) and EEG (generalized slow activity in those with encephalitis or cerebellitis). Age at infection was in two peaks, 1) in children aged 1 to 5 years (62% cases), and 2) in teenagers (24.6%). Febrile seizures occurred in 6 (3.1% of cases) <5 years age, and headaches in 47 (24.2%) patients, mostly teenagers 13-17 years old. Ten children presented with severe, neurologic complications: meningoencephalitis, acute encephalitis (5 cases), acute cerebellitis (2), transverse myelitis, and myeloradiculitis. Epstein-Barr virus is a common pathogen that should be tested for routinely in pediatric patients with a neuroinfection. [1]

COMMENTARY. The diagnosis of neurological complication of Epstein-Barr virus infection may be difficult to differentiate from febrile seizure in young children and from infectious mononucleosis in older children with headaches. The authors suggest that the incidence of neurologic pathology may be underestimated and the pathogenesis of Epstein-Barr virus encephalitis is often undetermined. Two previous reviews of pediatric Epstein-Barr virus associated encephalitis emphasize the difficulty in diagnosis, prognosis and the neuroanatomic localization of the infection. Twenty-one (6%) of 216 children in the Encephalitis Registry at University of Toronto had Epstein-Barr virus serology and/or positive PCR; 81% had CSF pleocytosis, 48% had seizures, 57% an abnormal EEG slow background, and 71% had abnormal MRI. Two patients died, 2 suffered mild deficits, and 16 were neurologically normal at follow-up. Only 1 had symptoms of infectious mononucleosis; all others had nonspecific prodrome including fever and headache [2]. In a review of 100 cases of Epstein-Barr virus encephalitis from University of Lund, Sweden, the neuroanatomic localization of involvement was the cerebral hemispheres, cerebellum and basal ganglia. Patients with isolated hemispheric gray or white matter involvement achieved good recovery while half of the patients with thalamic involvement developed sequelae. The highest mortality rate was among patients with isolated brain stem involvement. Neuroanatomic distribution of radiologic abnormalities in Epstein-Barr virus encephalitis may be a prognostic indicator [3].
